# Investigating the Formation and Molecular Solubilization Mechanism of Emodin Solid Dispersions by Molecular Dynamics Simulation

**DOI:** 10.3390/molecules30040822

**Published:** 2025-02-10

**Authors:** Jiaoyue Zhu, Haiju Bai, Shili Pan, Wei Shen, Jingying Li, Xuehui Ding, Lin Wang, Wei Xu

**Affiliations:** School of Pharmacy, Changchun University of Chinese Medicine, Changchun 130117, China; 15834878190@163.com (J.Z.); 933912337@139.com (H.B.); 15704470610@163.com (S.P.); 15124342946@163.com (W.S.); 18043910903@163.com (J.L.); 15837573625@163.com (X.D.)

**Keywords:** emodin, solid dispersions, molecular dynamics simulation, formation mechanism, solubilization mechanism

## Abstract

The preparation of solid dispersions (SDs) of emodin (EMO) represents an effective strategy for enhancing its limited water solubility. However, there is a lack of effective strategies for carrier screening. The molecular mechanism underlying EMO-SDs has yet to be fully elucidated. In this study, we employed a molecular simulation to identify the optimal solubilizing carriers for EMO-SDs, which were subsequently validated through solubilization experiments. Gelucire 50/13 (GEL) was identified as the most effective solubilizing carrier. The formulation of the EMO-SDs was established through solubility testing, utilizing a drug-to-carrier loading ratio of 1:9. The characterization of the interactions between the drug and the carrier was conducted using DSC, FTIR, and NMR spectroscopy. The DSC results indicated that EMO molecules were dispersed within the carrier in an amorphous state, while FTIR and NMR analyses revealed the formation of hydrogen bonds between the drug and carrier molecules. The molecular mechanisms of EMO-SDs were further elucidated through an MD simulation. Findings from the formation mechanism studies demonstrated that the majority of EMO molecules were embedded within the interstices of a loosely aggregated micelle-like structure formed by the carrier molecules. The solubility enhancement mechanism indicated that the carrier molecules surrounded the EMO molecules during the solubilization process, thereby facilitating the interaction of EMO with water. The stability mechanism accounts for the fact that recrystallization of the drug may occur.

## 1. Introduction

Emodin (EMO) is a naturally occurring compound that can be extracted from the roots and barks of various plants, including those of Rheum palmatum, Reynoutria japonica, and Zoysia japonica. It is also a fungal metabolite. Recent pharmacological research has demonstrated that EMO exhibits a diverse range of therapeutic benefits, including anti-inflammatory [1,2], antitumor [3], antibacterial, cardioprotective, neuroprotective [4], and nephroprotective properties. However, its limited water solubility and low bioavailability pose significant challenges to its clinical application. The formulation of insoluble drugs as solid dispersions (SDs) can effectively reduce the size of the drug crystals, inhibit their aggregation, and enhance the dispersion of drug molecules [5]. Therefore, this study aims to develop innovative SD drug delivery systems for EMO to improve its water solubility [6].

Drug–carrier compatibility is used to describe the miscibility of a drug–carrier system [7]. Theoretical considerations suggest that compatibility at the molecular level is crucial for attaining optimal physical stability in amorphous SDs [8]. The solubility parameter (δ) and the Flory–Huggins interaction parameters (χ) are frequently used to evaluate drug–carrier compatibility [9]. Lee et al. [10] calculated the δ between norfloxacin and various carriers to assess the drug–carrier compatibility. The optimal carrier identified for SDs of norfloxacin was hydroxypropyl methylcellulose phthalate (HPMCP). Sunita Metre [11] conducted calculations of χ between rivaroxaban and three different carriers in order to evaluate the compatibility of the drug with these carriers. The findings indicated that ES100 was the most suitable carrier for rivaroxaban SDs.

The interaction between the drug and the carrier is regarded as a critical factor influencing the physical stability of SDs [12]. Presently, the predominant methods employed to investigate the intermolecular interactions in SDs include Fourier transform infrared spectroscopy (FTIR), Raman spectroscopy, and nuclear magnetic resonance (NMR). Additionally, Differential Scanning Calorimetry (DSC) is a thermal analysis technique frequently employed to ascertain the physical state of a substance, which has increasingly been utilized for the characterization of SDs in recent years.

Molecular dynamics (MD) simulation is a widely used technique to explore the molecular mechanisms of SDs. Ouyang et al. employed an MD simulation to investigate the annealing process utilized in the formation of the molecular structure of ibuprofen SDs prepared via the hot-melt method [13]. Chan et al. utilized an MD simulation to investigate the dissolution process of ibuprofen SDs at the molecular level, thereby offering detailed insights into the dissolution mechanism [14].

In this study, we employed δ and χ for the first time to screen the excipients of EMO and subsequently validated them through solubilization experiments utilizing polymeric carriers. The SDs were prepared and assessed for drug–carrier interactions. The dispersion state of EMO within the carrier, as well as the mechanisms of solubilization and stabilization for EMO, were examined using MD simulations. This innovative approach, which integrates MD simulation technology with traditional experimental methods, provides both an empirical foundation and a theoretical framework for the development of other insoluble drug molecules into novel dosage forms.

## 2. Results

### 2.1. Solubility Parameter

In general, the compatibility of two substances can be assessed by the disparity in their δ. A smaller discrepancy in these parameters indicates a greater compatibility between the substances. The calculated δ values are presented in Table 1. The compatibility ranking of the drugs with their carriers is as follows: GEL > HPMC > PVPK30 > PVPVA64.

### 2.2. Flory–Huggins Interaction Parameter

The χ of drug–carrier can be calculated through Equation (3). As shown in Table 2, the compatibility order was as follows: EMO-GEL > EMO-HPMC > EMO-PVPK30 > EMO-PVPVA64. This order was consistent with the solubility parameter results. If χ is negative or approaches zero, the two components will exhibit miscibility, with a critical value of χ at 0.5, above which the components become partially miscible [15]. Based on this criterion, EMO demonstrates miscibility with GEL while exhibiting limited miscibility with the other carriers.

### 2.3. Solubility Studies with Polymer Carriers

As indicated in Table 3, EMO exhibited the highest solubility within a 1% GEL aqueous solution. The standard deviations calculated for all three measurements are within reasonable limits. Consequently, GEL was chosen as the carrier for SDs in subsequent investigations.

### 2.4. Dissolution Study Analysis

**Figure 1:**
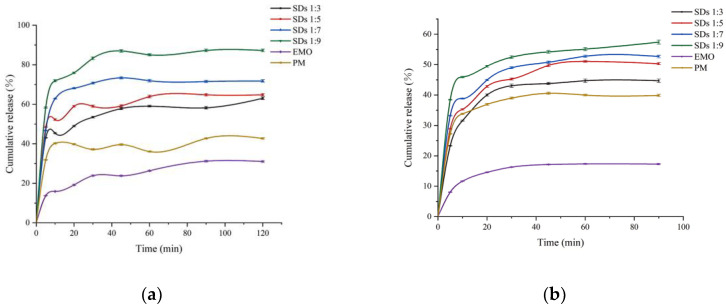
Dissolution profiles of EMO and formulations (n = 3) in (**a**) pH 6.80 buffer and (**b**) pH 1.2 buffer.

As illustrated in Figure 1a, the dissolution rate of SDs with a drug-loading ratio of 1:9 in phosphate buffer at pH 6.8 reached 85% at 60 min, which is approximately 2.8 times greater than that of the EMO. As illustrated in Figure 1b, the dissolution rate in hydrochloric acid solution at pH 1.2, where the dissolution rate of the SDs with a drug-loading ratio of 1:9 at 60 min was 55%, represents a 3.2-fold increase compared to EMO. The dissolution rates of the EMO-SDs were significantly higher than those of EMO when formulated as SDs utilizing GEL as a carrier. Furthermore, both EMO and its SDs exhibit increased solubility within the small intestinal environment. Among all tested drug-loading ratios, the dissolution rate of the EMO-SDs consistently demonstrated superior performance at a drug-loading ratio of 1:9 compared to other ratios. Consequently, a drug-loading ratio of 1:9 was determined to be the optimal formulation for subsequent investigations.

### 2.5. DSC Analysis

**Figure 2:**
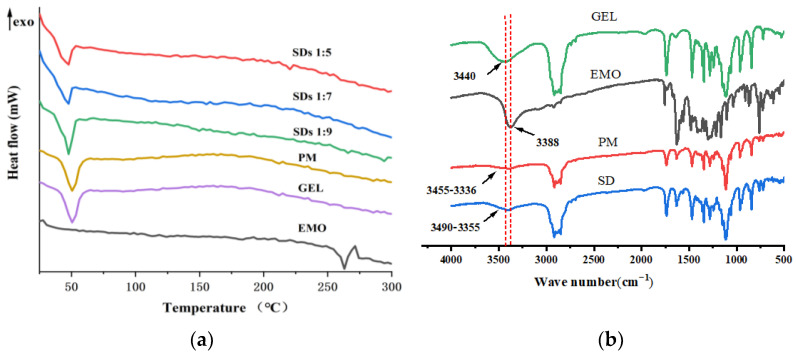
DSC thermograms (**a**) and FTIR spectra (**b**) of EMO, GEL, PM, and SDs.

As illustrated in Figure 2a, EMO exhibited a distinct exothermic reaction at 257 °C, serving as a reliable indicator for the crystal form of EMO. GEL, on the other hand, demonstrated a pronounced absorption peak at 50 °C, suggesting that its melting point is at 50 °C. Furthermore, no endothermic peak for EMO was observed in the SDs or PM, indicating that the drug was present in an amorphous state within the carrier.

### 2.6. FTIR Analysis

As illustrated in Figure 2b, the spectrum of EMO reveals a prominent peak at 3388 cm^−1^, which corresponds to the stretching vibration of phenolic hydroxyl groups, characterized by a distinct sharp peak [16]. The absorption peak observed at 3440 cm^−1^ in GEL is attributed to the stretching vibration of the N-H bond. In the case of PM, a broad and less pronounced absorption peak is noted at 3336~3455 cm^−1^, which is associated with the infrared absorption of O-H groups in EMO, linked through hydrogen bonding. In the SDs, a broad and intense absorption peak was recorded at 3355~3490 cm^−1^. When compared to EMO and PM, this peak exhibited a slight blue shift and an increased width, indicating an enhancement in bond energy. This phenomenon is speculated to arise from hydrogen bonding between the phenolic hydroxyl group of EMO and the N-H bond of the amide group within the carrier.

### 2.7. NMR Analysis

**Figure 3:**
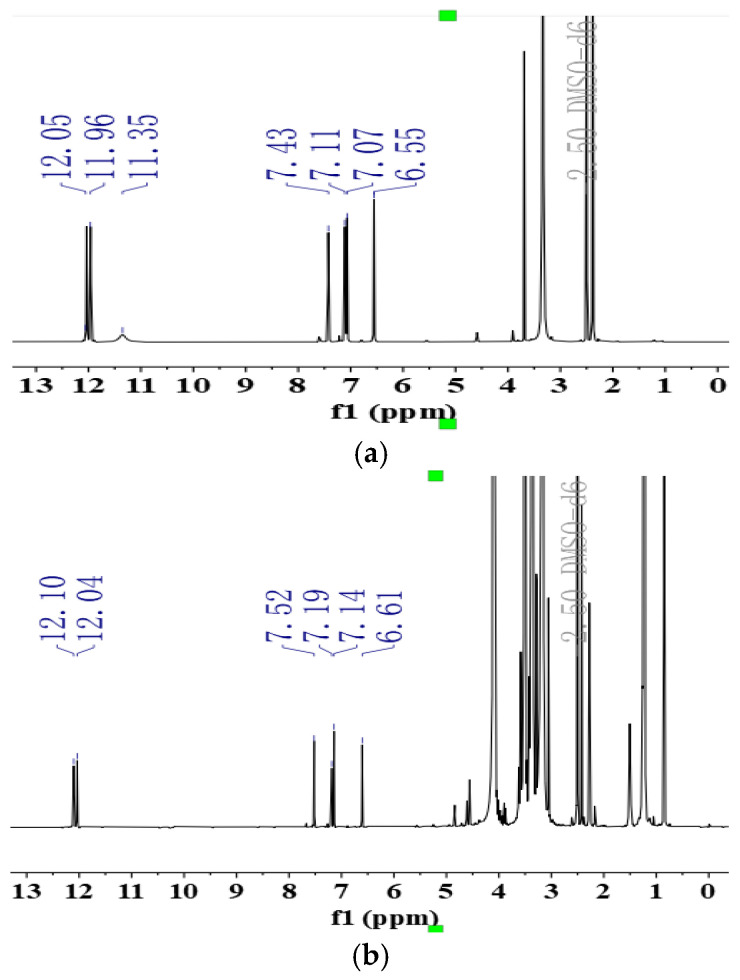

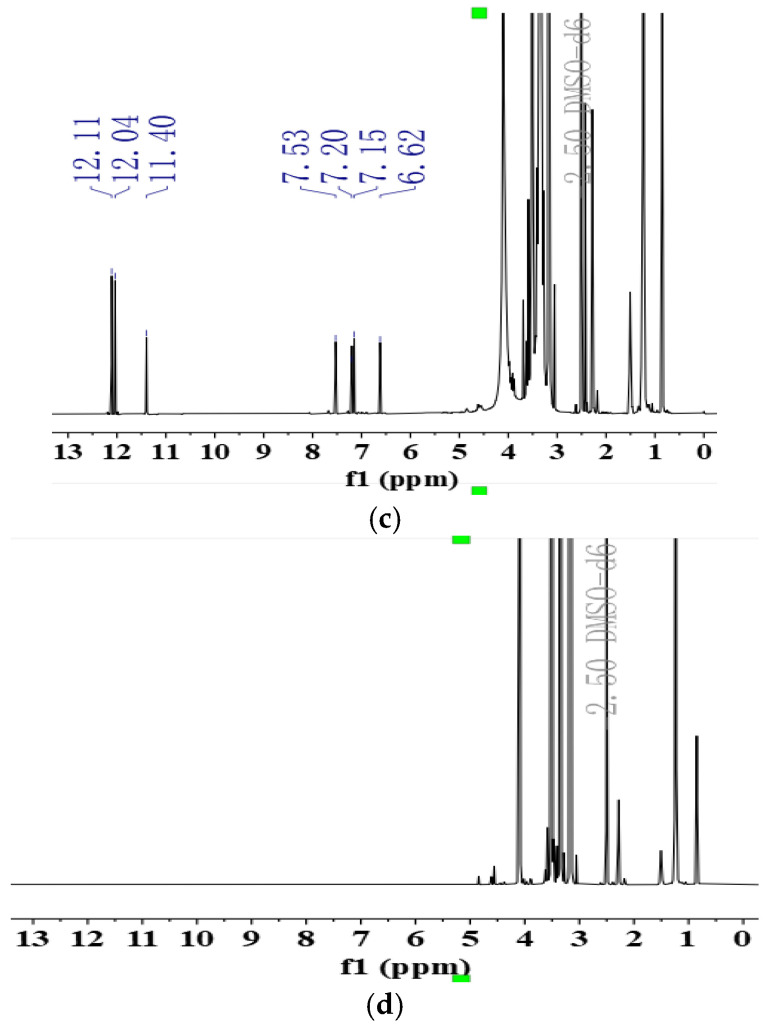
NMR spectrum of EMO (**a**), SD (**b**), PM (**c**), and GEL (**d**).

The detailed chemical shift values are presented in Table 4. As illustrated in Figure 3, the resonance peaks at δ 6.55, 7.07, 7.11, and 7.43 ppm correspond to the -CH- protons on the benzene ring of the drug, while the peaks at δ 11.35, 11.96, and 12.05 ppm are attributed to the phenolic hydroxyl group on the benzene ring of EMO. The resonance peak corresponding to the phenolic hydroxyl group at the third position on the benzene ring of EMO, initially observed at δ 11.35, vanished in the spectrum of the SDs [17]. It is speculated that EMO forms hydrogen bonds with GEL at the third position in the solution state. The phenolic hydroxyl group at this position serves as a hydrogen bond donor, while multiple amide groups within the carrier molecule act as hydrogen bond acceptors, facilitating the formation of these bonds.

### 2.8. Physical Stability of SDs

**Figure 4:**
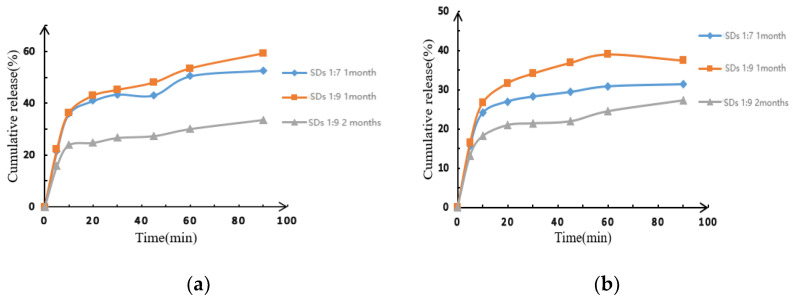
Dissolution curves for EMO-GEL-SDs with different drug-loading ratios at (**a**) pH 6.8 and (**b**) pH 1.2.

The SDs with varying drug-loading ratios exhibited moisture absorption and agglomeration after 1 month of storage under accelerated conditions at 40 °C and 75% relative humidity. Following this period, the SDs were removed, dried at room temperature, ground, and subsequently sieved through an 80-mesh sieve. Phosphate buffer at pH 6.8 and hydrochloric acid at pH 1.2 were selected as the dissolution media for assessing the cumulative dissolution of the SDs. The results indicated that the cumulative dissolution of the SDs with different drug-loading ratios demonstrated varying degrees of reduction, as illustrated in Figure 4. Notably, the cumulative dissolution of the SDs subjected to accelerated conditions at 40 °C and 75% humidity for two months exhibited a significant decrease compared to that of the SDs stored for 1 month. This finding suggests that the SDs are unstable when stored at 40 °C and 75% humidity, leading to aging and recrystallization of the drug.

### 2.9. Mechanism of SD Formation

**Figure 5:**
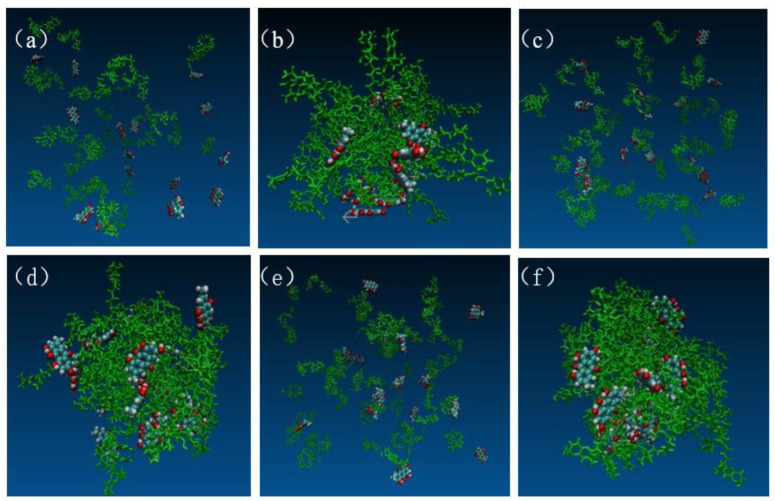
Snapshots of EMO-GEL-SDs; the initial structures of systems with different drug-loading ratios, (**a**) 1:5, (**c**) 1:7, (**e**) 1:9; the initial structures of simulated annealing with different drug-loading ratios, (**b**) 1:5, (**d**) 1:7, (**f**) 1:9.

The dynamic formation process of EMO-GEL-SDs was mapped by VMD, as shown in Figure 5. Figure 5a,c,e illustrate the initial structures of SDs constructed using Packmol for drug-loading ratios of 1:5, 1:7, and 1:9, respectively. Conversely, Figure 5b,d,f present the initial structures of SDs after undergoing simulated annealing for the same drug-loading ratios of 1:5, 1:7, and 1:9, respectively. Figure 5b,d,f distinctly reveal that all carrier molecules adopted irregular bends and folds, resulting in the formation of a loose micelle-like aggregate structure, within which all 16 EMO molecules were embedded or attached [18]. In Figure 5b, the carrier molecules are clustered together, with the majority of EMO molecules embedded within the inner space of the carrier aggregate structure. A minority of EMO molecules are dispersed on the surface of the carrier. Conversely, in Figure 5d, all carrier molecules are clustered, and the EMO molecules are more uniformly dispersed throughout the aggregated carrier structure compared to the 1:5 drug-loading ratio system. Most EMO molecules reside within the interstitial spaces of the aggregate, while only a few are present on the carrier structure’s surface. In Figure 5f, eleven EMO molecules were bound to the carrier molecules, while five remained in a free state. Following simulated annealing, the drug molecules were not uniformly dispersed within the carrier molecules, and some EMO molecules remained in close proximity to one another. This suggests a potential risk of drug recrystallization among adjacent EMO molecules during storage.

**Figure 6:**
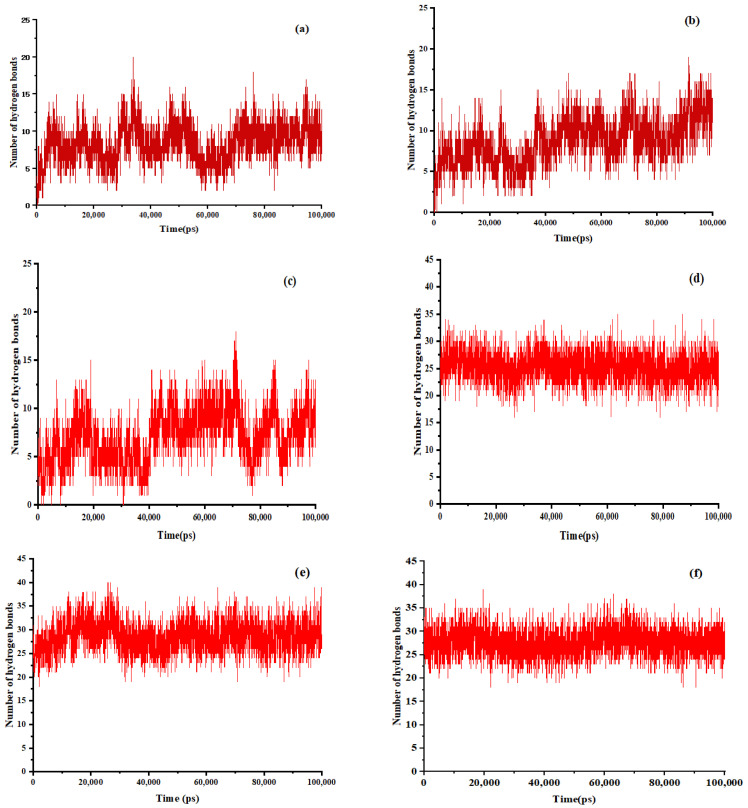
The number of hydrogen bonds between EMO and GEL in ethanol, (**a**) drug-loading ratio of 1:5, (**b**) drug-loading ratio of 1:7, (**c**) drug-loading ratio of 1:9; the number of hydrogen bonds between EMO and GEL in a 100 ns MD simulation process after simulated annealing, (**d**) drug-loading ratio of 1:5, (**e**) drug-loading ratio of 1:7, (**f**) drug-loading ratio of 1:9.

Hydrogen bonding is a crucial force in the binding process. Figure 6 illustrates the fluctuation in the quantity of hydrogen bonds throughout the system’s simulation. During the dissolution process in anhydrous ethanol, a specific quantity of hydrogen bonds was established between EMO and GEL. Following the heating and subsequent evaporation of the anhydrous ethanol, an increased number of hydrogen bonds formed between the drug molecules and the carrier molecules, surpassing the quantity observed in the ethanol solution. The findings indicated that EMO molecules and GEL molecules interact through the formation of hydrogen bonds. Furthermore, the system with a drug-loading ratio of 1:9 exhibited a greater number of hydrogen bonds compared to those with drug-loading ratios of 1:5 and 1:7. This finding substantiates the enhanced stability of the SDs at a 1:9 drug-loading ratio and reaffirms the accuracy of the previously determined optimal drug-loading ratios.

**Figure 7:**
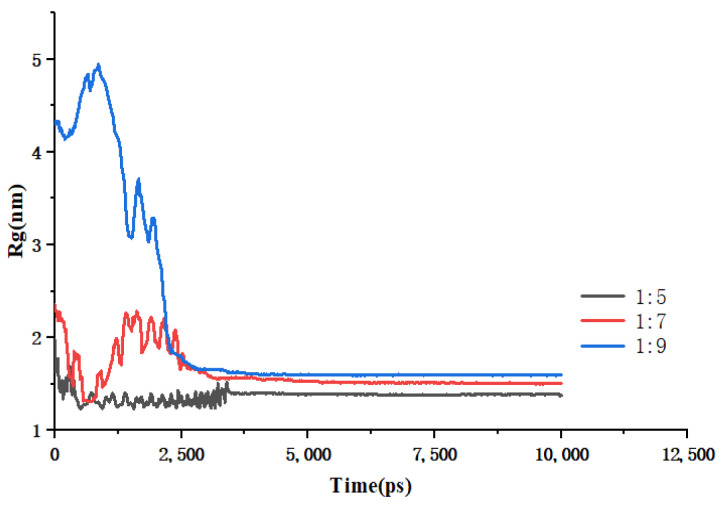
Changes in Rg over time during simulated annealing of EMO-GEL-SDs.

The calculation of the radius of gyration (Rg) between the drug molecule and the carrier molecule facilitates the assessment of the binding affinity between the two entities. Figure 7 illustrates the results of the Rg for various drug–carrier ratio systems throughout the annealing process as a function of time. It is observed that the radius of gyration between the drug and the carrier diminishes to a smaller value with an increase in annealing time. These findings suggest that the removal of ethanol during the cooling process to room temperature results in a strong binding interaction between the drug molecules and the carrier molecules.

### 2.10. Dissolution Enhancement Mechanism of SDs

**Figure 8:**
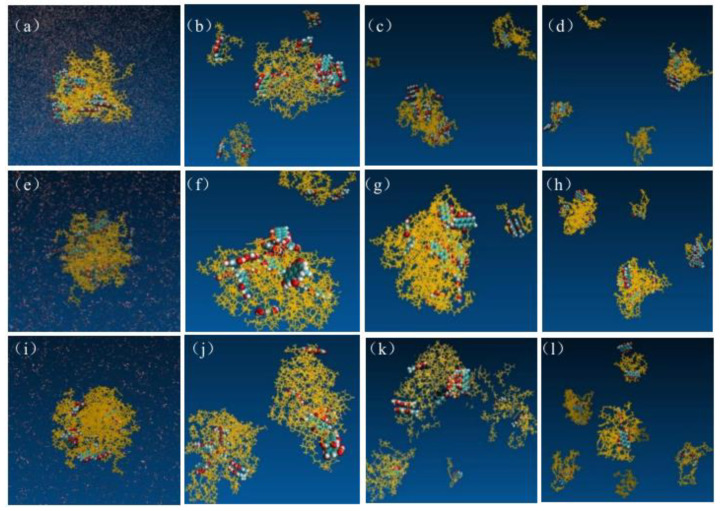
Structures of EMO-GEL SDs with a 1:5 drug-loading ratio in water, (**a**) 0 ns, (**b**) 64 ns, (**c**) 200 ns, (**d**) 400 ns; structures of EMO-GEL SDs in water with a 1:7 drug-loading ratio, (**e**) 0 ns, (**f**) 50 ns, (**g**) 200 ns, (**h**) 370 ns; structure of EMO-GEL SD with a 1:9 drug-loading ratio in water, (**i**) 0 ns, (**j**) 50 ns, (**k**) 300 ns, (**l**) 600 ns.

Figure 8 illustrates the distinct molecular images depicting the dissolution process of EMO-GEL-SDs with varying drug-loading ratios in water. Images were made by the VMD software Version 1.9.4. During dissolution, the relaxation state of micelle-like aggregates gradually increases with time, and more and more EMO molecules are gradually released from the SD system. As drug molecules diffused into the aqueous solution, they were perpetually encircled by carrier molecules. The dissolution of these carrier molecules facilitated the interaction between EMO and water. This is precisely the rationale behind the enhanced water solubility of EMO observed as a result of the preparation of EMO-GEL-SDs. The segments (b–d), (f–h), and (j–l) in Figure 8 demonstrated that the drug molecules progressively dissolved into clusters of four to six, maintaining an amorphous state within the undissolved particles [19].

**Figure 9:**
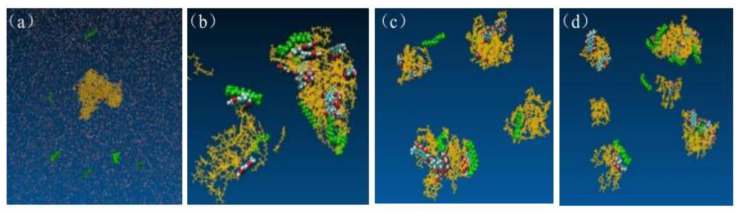
Structure of EMO-GEL-SDs with a drug-loading ratio of 1:9 in water containing SDS, (**a**) 0 ns, (**b**) 13 ns, (**c**) 64 ns, (**d**) 113 ns.

Figure 9 presents a simulation of the system, featuring a drug–carrier ratio of 1:9, within an aqueous solution that comprises 0.5% SDS. As depicted in Figure 9b, SDS molecules swiftly adhere to both EMO and carrier molecules. Moving to Figure 9c, the entire system undergoes dissolution, resulting in the formation of four to six smaller, loosely arranged fragments. Notably, this occurrence precedes that observed in all systems devoid of SDS. Subsequently, in Figure 9d, these reduced fragments persist in an unaggregated state. This observation offers a compelling theoretical foundation for the improved dissolution properties exhibited by the SDs when SDS is introduced into the system.

**Figure 10:**
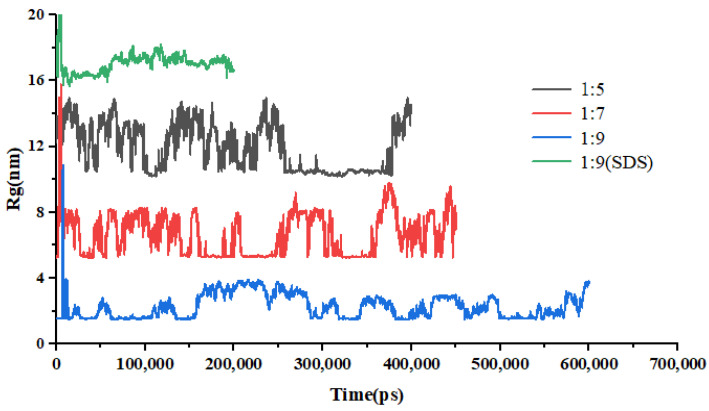
Changes in Rg of EMO-GEL-SDs with different drug-loading ratios over time.

The results pertaining to Rg across various systems as a function of simulation time are presented in Figure 10. The images were made using the VMD software. This figure illustrates that the radius of gyration for the EMO-GEL system exhibits fluctuations within a defined range centered on the X-axis. This behavior suggests that the drug molecules are encapsulated by the carrier molecules throughout the dissolution process. When considered alongside the detailed depiction of the dissolution process in Figure 10, it becomes evident that the carrier molecules inhibit the aggregation of drug molecules during dissolution.

### 2.11. Molecular Stability Mechanisms

**Figure 11:**
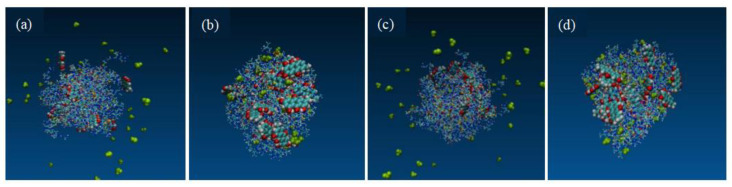
The system with a 1:7 drug-loading ratio; the initial structure is depicted (**a**); the final structure following a 400 ns MD simulation under simulated acceleration conditions (**b**). The system with a 1:9 drug-loading ratio; the initial structure (**c**); the final structure after a 400 ns MD simulation under similar simulated acceleration conditions (**d**).

Figure 11a,c illustrate the initial structures of the SDs formation process simulated via the solvent evaporation method, while (b) and (d) depict the final structure following a 400 ns simulation under accelerated conditions at 40 °C and 75% humidity. The images were made using the VMD software. During the preparation process, the crystalline drug transitions to an amorphous state, with the EMO molecules becoming embedded within the internal structure of the carrier molecules, resulting in the formation of looser micelle-like aggregates. In comparison to the initial structure, the carrier GEL, which exhibits high hygroscopicity, adsorbs nearly all available water molecules, leading to the development of a denser SD structure after the 400 ns simulation under accelerated conditions. This denser structure increases the distance between the drug molecules, thereby complicating the embedding of EMO molecules within the gaps of the carrier structure and facilitating the recrystallization of the drug molecules. Notably, the 1:9 SD formulation exhibited less aggregation of drug molecules than the 1:7 SD formulation. This phenomenon may be attributed to two factors: firstly, hydrogen bonding between the drug and the polymer carrier inhibits drug aggregation and recrystallization during storage; secondly, the spatial hindrance presented by the polymer restricts the movement of the drug molecules.

**Figure 12:**
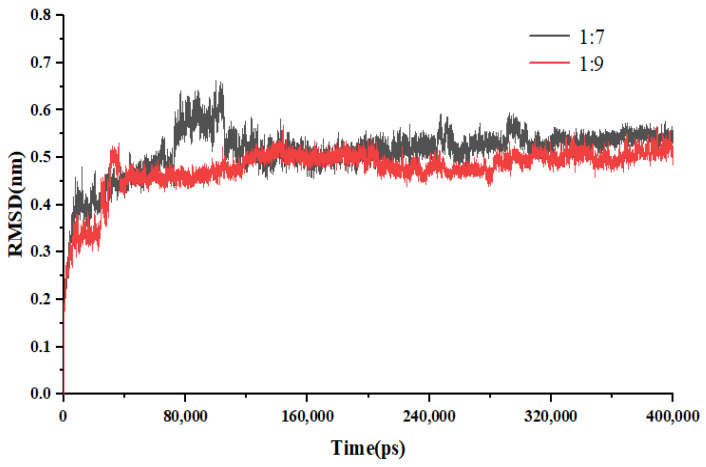
RMSD for accelerated testing of EMO-GEL-SDs.

Figure 12 illustrates the RMSD of the two systems throughout the 400 ns MD simulation conducted in the accelerated experiment. The data indicate that the drug-loading ratio of 1:7 SDs fluctuates at 4.5 Å, whereas the fluctuation observed for the 1:9 SDs is only 2 Å. This reduced fluctuation suggests that the 1:9 SD demonstrates greater stability compared to the 1:7 SD under conditions of high humidity and elevated temperature.

**Figure 13:**
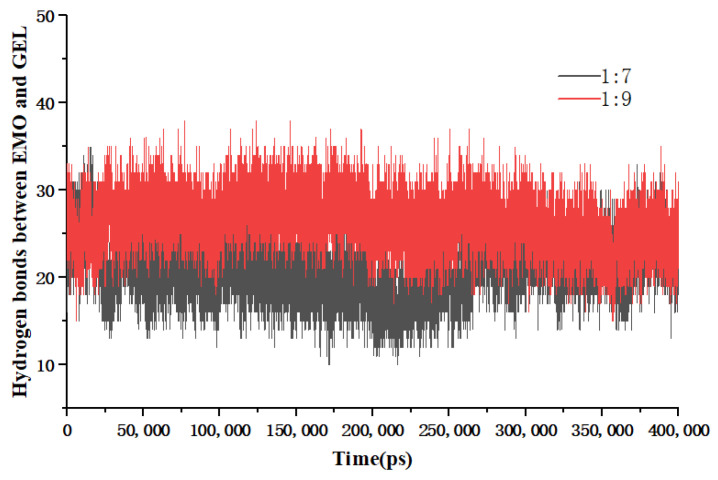
Number of EMO-GEL hydrogen bonds for accelerated testing.

Figure 13 illustrates the temporal variation in the number of hydrogen bonds between EMO and GEL throughout the simulation. The data indicate that the 1:9 SD exhibits a greater number of hydrogen bonds compared to the 1:7 SD. This finding supports the hypothesis that hydrogen bonding plays a crucial role in inhibiting the aggregation and recrystallization of EMO molecules during storage.

## 3. Discussion

SDs serve as a crucial formulation technique to enhance the dissolution rate and biological attributes of insoluble compounds. The solubility of crystalline medications within polymers, as well as the miscibility of amorphous drugs within polymeric matrices, are intimately tied to the stability of these drugs [20]. Therefore, researchers must meticulously evaluate the solubility and miscibility of drugs within polymers, aiming to select the most suitable and optimal formulation, encompassing polymer type, drug loading, and other relevant factors, as well as appropriate storage conditions, to ensure maximum physical stability of the drug. In this study, the δ and χ were used to screen the excipients compatible with EMO. The δ and χ were calculated using an MD simulation, and the results showed that the excipient with the best compatibility with EMO was GEL.

Exploring the essence of amorphous SDs (ASDs) and evaluating the inherent danger of recrystallization in ASDs necessitates the comprehensive characterization of the formulations. Adhering to the quality by design principle, researchers must possess a profound comprehension of the molecular-level processes that unfold [21]. No single method of characterization can yield a comprehensive understanding; hence, a blend of various characterization tools is imperative. The commonly employed approaches for the characterization of SDs encompass dissolution, X-ray diffraction (XRD), NMR, FTIR, and DSC. In the current investigation, solubility served as a pivotal indicator, and the optimal formulation ratio was identified as a drug-to-carrier ratio of 1:9. The results of DSC showed that no heat absorption peak of EMO was detected in both SDs and PM, indicating that the drug was dispersed in the carrier in an amorphous or molecular form. The FTIR tests inferred that the phenolic hydroxyl group in EMO interacts with the nitrogen–hydrogen bond of the amide group in the carrier by hydrogen bonding. The NMR experiments have inferred that EMO forms hydrogen bonds with GEL at position 3 in the solution state, with the phenolic hydroxyl group at position 3 acting as a hydrogen bond donor, and multiple amide groups in the carrier acting as hydrogen bond acceptors. The primary goal of formulating SDs is to uniformly disperse the drug within the carrier matrix, enabling the hydrophilic carrier to encapsulate the hydrophobic drug. This encapsulation ensures the enhanced wettability of the drug, ultimately augmenting its in vitro dissolution rate.

In the current study, the process of forming SDs of the bioactive molecule EMO was simulated utilizing an MD simulation. Subsequently, the initial structure post-annealing was visualized through Visual Molecular Dynamics (VMD), revealing that EMO exists in the carrier in a molecular configuration, with a notable reduction in its crystallinity. The carrier molecule comprises amide groups, which are capable of forming robust hydrogen bonds with the phenolic hydroxyl groups present on the EMO molecule. These potent drug–carrier interactions lead to the disruption of EMO’s original crystalline structure. Furthermore, the intricate intertwining between EMO and the carrier facilitates the complete dispersion of the EMO molecule, while the established hydrogen-bonding network enhances the drug’s molecular dispersion within the carrier.

Pranav et al. successfully prepared SDs of GEL with tacrolimus, observing that the GEL carrier effectively enhanced the dissolution rate of tacrolimus by reducing its crystallinity [22]. Chan et al. utilized an MD simulation to delve into the molecular dissolution mechanism of binary SDs. Their findings revealed that the particles of SDs composed of piroxicam and ibuprofen were amorphous in their undissolved state, whereas ibuprofen–polyethyleneglycol and ibuprofen–polyvidone particles exhibited aggregation of the drug molecules at a later stage of the dissolution [14]. This observation aligns with the conclusions drawn in this paper, emphasizing the efficacy of amphiphilic polymers, such as GEL, Poroxam, or HPMCAS, in inhibiting aggregation during dissolution and, thereby, enhancing the bioavailability of water-soluble drugs in SDs. In the current investigation, it was observed that the insoluble EMO molecules were encapsulated by water-soluble GEL molecules. This dissolution of GEL molecules facilitated the interface between EMO and water, while the carrier molecules significantly enhanced the wettability of the drug molecules. Observed during the dissolution of SDs, the drug, being highly dispersed, was encapsulated by a sufficient amount of carrier molecules. This encapsulation mitigated the likelihood of drug molecule aggregation, thus preserving the drug’s dispersed state and enhancing its dissolution rate. The investigation into the formation mechanism revealed that hydrogen bonds were established between drug molecules and carrier materials. These robust hydrogen-bonding interactions hindered the emergence and development of drug nuclei, thereby allowing EMO to be dispersed within the carrier materials in an amorphous, non-crystalline state. Consequently, the dissolution rate of the drug was enhanced.

This study presents certain limitations. Notably, a stability test at room temperature was not conducted, and the stability of the solid dispersions of EMO was solely evaluated through accelerated testing. Furthermore, the solid dispersions of EMO were examined exclusively in relation to their preparation and molecular mechanisms, with no in vivo pharmacological experiments undertaken. A comprehensive understanding of the absorption mechanisms and pharmacokinetic properties of EMO solid dispersions is essential. Future pharmacological investigations should prioritize the assessment of the pharmacological properties of EMO to provide robust support for its clinical applications.

## 4. Materials and Methods

### 4.1. Materials

EMO, Hydroxypropyl Methyl Cellulose (HPMC), Vinylpyrrolidone-vinyl acetate copolymer (PVPVA64), and Polyvinylpyrrolidone (PVPK30) were purchased from Shanghai Yuanye Biotechnology Co., Ltd. (Shanghai, China). Gelucire 50/13 (GEL) was presented by Gattefosse, France. KH_2_PO_4_ and NaOH were purchased from Tianjin Tianli Chemical Reagent Co., Ltd. (Tianjin, China). Hydrochloric acid (HCl) was purchased from Chengdu Kelong Chemical Co., Ltd. (Chengdu, China). Sodium dodecyl sulfate (SDS) was purchased from Beijing Jintai Hongda Biotechnology Co. Ltd. (Beijing, China). All other chemicals and reagents were of analytical or high-performance liquid chromatography (HPLC) grade.

### 4.2. MD Simulation Method for Solubility Parameter

The δ is indicative of a drug’s solubility within a polymer. It quantifies the square root of the cohesive energy density (CED), which represents the energy needed per unit volume to disrupt the intermolecular forces and vaporize one mole of molecules from the liquid state (Equation (1)) [23]. The molecular structure of EMO underwent structural optimization on four separate occasions. This optimization process was conducted with ultra-fine quality, utilizing the COMPASS force field and incorporating automatic charge assignment. Following this, a cubic cell composed solely of EMO molecules was constructed. This cell underwent structural optimization three additional times with ultra-fine precision. Subsequently, an MD simulation was performed. Upon completion of the MD simulation, δ calculations were conducted. To ensure the reliability of the simulated data, these calculations were repeated three times, resulting in an average δ value. The same methodology was applied to calculate the δ values for PVPK30, PVPVA64, HPMC, and GEL, respectively. Subsequently, the disparity in the solubility parameter between the drug and its carrier was determined using Equation (2).

(1) $$\delta ={\left(CED\right)}^{1/2}{\left(E∕V\right)}^{1/2}$$

(2) $$∆\delta =|\delta_{A}-\delta_{B}|$$

where δ is the solubility parameter value, E represents the evaporation energy, V is the molar volume of the liquid at the evaporation temperature, and δA and δB represent the δ values of the drug and polymer, respectively.

### 4.3. Flory–Huggins Interaction Parameter

The calculation of χ has been established as a method for the quantitative assessment of miscibility in amorphous drug–polymer systems [24]. The value of χ, which represents the interaction parameter between the drug and the polymer, is a critical determinant of whether spontaneous mixing can occur. Typically, when χ is either negative or a small positive value, the strength and extent of the interactions between the components are primarily attributed to a single component, indicating compatibility between the two components. Conversely, when χ assumes a large positive value, it suggests that the two components exhibit difficulty in mixing at the molecular level [16]. Consequently, the investigation of χ is essential for understanding and predicting the behavior of drug/polymer systems. The value of χ can be determined through two approaches: the solubility parameter method and the melting inhibition method. In this study, we employed the solubility parameter method to calculate the χ value, as shown in Equation (3).

(3) $$\chi =V∕RT{\left(∆\delta \right)}^{2}$$

where δ represents the value of the solubility parameter, R is the gas constant, T is the absolute temperature, and V represents the molecular volume of the drug, obtained by dividing the molecular molar mass by the density. The resulting χ can be used to make a preliminary determination of the compatibility of the two components.

### 4.4. Solubility Studies with Polymer Carriers

In order to validate the results of the drug carrier compatibility screening, we prepared 1% aqueous solutions by incorporating precise quantities of PVPK30, PVPVA64, HPMC, and GEL into a hydrochloric acid solution, simulating gastric fluid at pH 1.2, as well as into phosphate buffer, simulating small intestine fluid at pH 6.7 [25]. An excess of EMO was subsequently added to the 1% aqueous solution, which was agitated at 120 rpm and maintained at 28 °C for a duration of 48 h. Following this period, the solution was subjected to centrifugation at 1000 rpm for 10 min, after which the supernatant was filtered using a 0.45 μm water filtration membrane. The concentration of the components was quantified using HPLC (Shimadzu, Kyoto, Japan). All experiments were conducted in triplicate.

### 4.5. Preparation of SDs

EMO and GEL were combined in various mass ratios (1:3, 1:5, 1:7, and 1:9) and subsequently dissolved in anhydrous ethanol. The solvent was then evaporated by heating in a water bath maintained at 50 °C. Following this process, the samples were dried, ground, and subsequently sieved through an 80-mesh screen [26]. The resulting SD powder was stored in a desiccator.

### 4.6. Preparation of Physical Mixture (PM)

The EMO and GEL samples, maintained at a mass ratio of 1:9, were precisely weighed and then transferred into a mortar. The samples were thoroughly ground to achieve a uniform mixture. Subsequently, the homogenized mixtures were passed through an 80-mesh sieve and stored in a desiccator for preservation.

### 4.7. Dissolution Tests

The in vitro dissolution studies were conducted in a 708-DS dissolution apparatus (Agilent Technologies, CA, USA) using the paddle method. The solution of 900 mL of HCl with pH 1.2 was used to simulate gastric fluid, and 900 mL of phosphate buffer with pH 6.8 was used to simulate small intestinal fluid, as well as 0.5% SDS was added as the dissolution medium, respectively. The samples of EMO, SDs, and PM, each containing 20 mg of EMO, were placed into the dissolution medium at 37(±0.5) °C with a rotation speed of 100 rpm. The paddle method was adopted to conduct the dissolution test. Aliquots of 2 mL were systematically collected at intervals of 5, 10, 20, 30, 45, 60, 90, and 120 min. During each sampling event, an equivalent volume of pre-heated medium was replenished to sustain a constant volume. The collected samples were subsequently filtered through a 0.45 μm water filter, diluted with 1:2 methanol, and then entered into a high-performance liquid chromatography system (Nexis GC-2030, Kyoto, Japan) for analysis.

### 4.8. Differential Scanning Calorimetry (DSC)

DSC (Mettler Toledo, Zurich, Switzerland) was used for a thermal analysis. DSC is a widely utilized thermal analysis technique that is employed to determine the phase transitions or forms of various substances. In the present experiment, samples of EMO, GEL, PM, and SDs were subjected to a temperature scan ranging from 25 °C to 300 °C within a nitrogen atmosphere. The volumetric flow rate of nitrogen was maintained at 50 mL/min, and the heating rate was established at 10 °C/min. The onset temperature of the melting endothermic peak was recorded for each sample.

### 4.9. Fourier Transform Infrared Spectroscopy (FTIR)

FTIR spectra are instrumental in examining the interactions between drug and excipient molecules. The FTIR spectra were recorded using an FTIR spectrometer (Vertex 70v, Bruker, Karlsruhe, Germany). A small sample of each component (EMO, GEL, PM, and SDs) was mixed with an appropriate amount of KBr, ground thoroughly, and then compressed into a transparent disc. The KBr discs were subsequently analyzed using an infrared spectrometer, with a spectral scanning range of 400–4000 cm^−1^, an instrument resolution of 4 cm^−1^, and scanning times of 16 times.

### 4.10. Nuclear Magnetic Resonance (NMR) Spectroscopy

NMR spectroscopy is employed to examine the interactions between pharmaceutical compounds and polymer carriers in solutions. The substances EMO, GEL, PM, and SDs were analyzed using ^1^H-NMR spectroscopy. Specifically, 5 mg of each substance—EMO, GEL, PM, and SDs—was accurately measured and weighed. Following this, 0.6 mL of deuterated DMSO was utilized to dissolve each sample, and the resulting solutions were subsequently transferred into a 5 mm diameter NMR tube for further characterization. Tests were carried out using an NMR spectrometer (Avence III 600, Bruker, Karlsruhe, Germany), acquiring a zg 30 pulse sequence to determine the ^1^H-NMR of the samples at 298 K, with 16 scans.

### 4.11. An Investigation into the Physical Stability of SDs

The SDs of EMO were subjected to a pharmaceutical synthesis and a stabilization assessment. The SDs of EMO were placed in the pharmaceutical synthesis and stabilization test chamber. The SDs were maintained under accelerated conditions at 40 °C and 75% relative humidity for a duration of 2 months. Samples were collected at one and two months to evaluate the in vitro dissolution of the SDs. A phosphate-buffered saline solution with a pH of 6.8, containing 0.5% SDS, was utilized as the dissolution medium, employing the paddle method in a volume of 900 mL. The dissolution process for the SDs, equivalent to 2 mg of EMO, was conducted at a temperature of 37 ± 0.5 °C, with a stirring speed of 100 r/min using the paddle apparatus. At intervals of 5, 10, 20, 30, 45, 60, and 90 min, 2 mL of the solution was withdrawn and immediately filtered through a 0.45 μm aqueous filter. An equivalent volume of the dissolution medium was replenished after each sampling. The filtered solution was subsequently diluted in a 1:1 ratio with methanol and injected in a volume of 20 μL for a cumulative dissolution analysis.

### 4.12. Formation Mechanism of SDs

The computer simulation work in this study was performed through the open-source MD simulation software GROMACS 2018.8 and the Generalized Amber Force Field (GAFF), which is common to all carriers and drugs. The main MD simulation software used included Packmol 18.169, VMD 1.9.3, Gaussian 09, Discovery Studio 2016 client, and AmberTools23. The initial configurations of all systems were constructed employing Packmol. The MD simulation method was used to simulate the preparation of SDs by solvent evaporation [27]. The initial structure was submerged in ethanol to a depth of 100 Å to create a solvation system, replicating the dissolution process of drugs and carriers within ethanol. During the minimization phase, two-stage least squares was employed to relax the solvent box. Initially, the solvent molecules underwent a 10,000-step steepest descent method minimization, followed by a 10,000-step conjugate gradient minimization to eradicate any improper interactions within the system’s molecules. After the energy minimization phase, the solvated system, which was subject to weak constraints, was gradually heated to a target temperature of 323 K. The temperature coupling was achieved using the velocity rescaling (v-rescale) method, incorporating a stochastic term. A total of 500 ps of the MD simulation were executed. Subsequent to the heating phase, the entire system underwent an MD simulation for 100 ns at a stable temperature of 323 K, with a time step of 2 fs per step and a cut-off radius for nonbonding interactions set at 10 Å. Following this, the ethanol molecules were extracted from the system, and a 10 ns simulated annealing process was conducted to gradually reduce the temperature from 323 K to 298.15 K. Ultimately, the simulated trajectory was subjected to analysis. Details of the simulation are shown in Table 5.

### 4.13. Dissolution Enhancement Mechanism of SDs

GROMACS 2018.8 and GAFF were employed to simulate the molecular dissolution mechanism [28]. The initial structure of the SDs, which was generated through a simulated annealing process, was subsequently submerged in a cubic TIP3P water box measuring 20 nm per side. Following the construction of the system with Packmol, the energy of the system was minimized to eradicate any improper molecular interactions. This energy minimization was executed via 10,000 steps of the steepest descent gradient minimization, succeeded by 10,000 steps of the conjugate gradient algorithm.

The entire mixed system was simulated under constant temperature and pressure conditions of 298.15 K and 1 atm, respectively. The step size is set to 2 fs. The range of van der Waals forces and electrostatic forces is set to 0.8 nm. Temperature regulation was achieved through the velocity-rescale method. Pressure coupling with the Berendsen method was performed while keeping the structure of the SDs under weak constraints. Simulations spanning 400 and 500 ns were conducted within a solvation environment to mimic the dissolution process of SDs in water. Details of the simulation are shown in Table 6.

### 4.14. Methods for Molecular Mechanisms of Stability

Simulations of molecular stability mechanisms were conducted utilizing GROMACS 2018.8. Molecular modeling techniques were employed to explore the molecular mechanisms underlying the stability of SDs at the molecular level. To replicate the accelerated conditions present in stability experiments, the quantity of water molecules and the specific simulation parameters are detailed in Table 7. The initial structures of SDs, with drug-loading ratios of 1:7 and 1:9, were generated through simulated annealing and subsequently combined with water molecules using the Packmol procedure. Each system was initially heated to 313 K and then subjected to a simulation duration of 400 ns. The systems were subsequently analyzed for RMSD, intrinsic contact points, and number of hydrogen bonds. RMSD quantifies the average deviation of atoms from their initial configurations. A critical distance of 3 Å was established to define the natural contact points between EMO and GEL molecules, indicating the aggregation of EMO molecules. Furthermore, the influence of the number of hydrogen bonds—both among the drug molecules and between the drug and the carrier—on the stability of SDs was assessed.

## 5. Conclusions

In this study, we first investigated the compatibility of EMO with various carriers using δ and χ. Subsequently, these compatibility assessment results were confirmed by solubilization experiments involving polymeric carriers. The optimum carrier for the SDs of EMO was screened as GEL. The optimum drug-accommodation ratio of 1:9 was screened by solubility experiments. The drug–carrier interactions were evaluated by FTIR, NMR, and DSC after preparation. It was found that EMO was dispersed in the carrier in an amorphous state, where the phenolic hydroxyl group of EMO acted as a hydrogen bond donor, and multiple amide groups in GEL acted as hydrogen bond acceptors to form hydrogen bonds. In addition, the dispersion state of EMO in the carrier and the solubilization mechanism of EMO by the carrier were also investigated by an MD simulation, and the stabilization mechanism of EMO SDs was analyzed.

In this research endeavor, an MD simulation was harnessed for the screening of SD carriers, while polymer carrier solubilization experiments were employed to validate the predictive outcomes pertaining to drug-carrying compatibility. This integrated approach has yielded a comprehensive suite of rapid and precise carrier screening tools, offering methodological versatility for the fabrication of diverse SDs. Furthermore, the MD simulation was leveraged to delve into the intricacies of the formation mechanism and solubility enhancement mechanisms of EMO SDs. These simulations provided invaluable insights into the behavioral patterns of small molecules.

The strategic alliance between traditional experimental methodologies and MD simulations represents an efficacious paradigm for tackling the pivotal challenges associated with SDs. This collaborative framework expedites the development of SD formulations for insoluble drugs.

## Figures and Tables

**Table 1:** Solubility parameter (δ) calculation results.

Substances	δ (J/cm^3^)^1/2^	Δδ (J/cm^3^)^1/2^
EMO	25.65	—
PVPK30	21.66	3.99
PVPVA64	20.14	5.51
HPMC	21.98	3.67
GEL	26.81	1.16

**Table 2:** Flory–Huggins (F-H) interaction parameter (χ) calculation results.

Drug–Carrier	χ
EMO-PVPK30	1.10
EMO-PVPVA64	2.09
EMO-HPMC	0.93
EMO-GEL	0.09

**Table 3:** Solubility of EMO in aqueous polymer carrier solutions.

Carrier Type	pH Value	Solubility (μg/mL)	Standard Deviation	Confidence Limits
-	1.2	-	-	-
-	6.8	-	-	-
PVPVA64	1.2	0.49 *	0.025	0.441–0.539
PVPVA64	6.8	2.38 *	0.0455	2.291–2.469
PVPK30	1.2	0.06 *	0.00163	0.0568–0.0632
PVPK30	6.8	0.37 *	0.0125	0.3455–0.3945
GEL	1.2	18.95 *	0.082	18.789–19.111
GEL	6.8	110.86 *	0.499	109.882–111.838
HPMC	1.2	-	-	-
HPMC	6.8	-	-	-

* Compared to EMO, *p* < 0.05 confidence level α = 0.05.

**Table 4:** ^1^H NMR spectrum information of EMO, SD, PM, and carrier (25 °C).

Sample	δa	δb	δc	δd	δe	δf	δg
EMO	6.55	7.07	7.11	7.43	11.35	11.96	12.05
SDs	6.61	7.14	7.19	7.52	—	12.04	12.10
PM	6.62	7.15	7.20	7.53	11.40	12.04	12.11

**Table 5:** Simulation details of SD systems.

SD System	Weight Ratio	Molar Ratio	Number of EMO Molecules	Number of GEL Molecules	Number of Ethanol Molecules	Total Atoms in Systems
EMO/GEL	1:5	16:36	16	36	8000	75,216
EMO/GEL	1:7	16:51	16	51	8000	76,356
EMO/GEL	1:9	16:64	16	64	10,000	95,344

**Table 6:** Simulation details of the dissolution process of SD systems.

SD System	Weight Ratio	Molar Ratio	Number of EMO Molecules	Number of GEL Molecules	Number of SDS Molecules	Number of Water Molecules	Total Atoms in System
EMO/GEL	1:5	16:36	16	36	0	30,000	93,216
EMO/GEL	1:7	16:51	16	51	0	30,000	94,356
EMO/GEL	1:9	16:64	16	64	0	20,000	65,344
EMO/GEL/SDS	1:9	16:64	16	64	7	20,000	65,638

**Table 7:** Simulation details of the molecular mechanisms of stability of SD systems.

SD Systems	Weight Ratio	Molar Ratio	Number of EMO Molecules	Number of GEL Molecules	Number of Water Molecules	Total Atoms in System
EMO/GEL	1:7	16:36	16	51	50	4506
EMO/GEL	1:9	16:64	16	64	50	5494

## Data Availability

Data are contained within the article.
